# Image-to-Patient Registration in Computer-Assisted Surgery of Head and Neck: State-of-the-Art, Perspectives, and Challenges

**DOI:** 10.3390/jcm12165398

**Published:** 2023-08-19

**Authors:** Ali Taleb, Caroline Guigou, Sarah Leclerc, Alain Lalande, Alexis Bozorg Grayeli

**Affiliations:** 1 Team IFTIM, Institute of Molecular Chemistry of University of Burgundy (ICMUB UMR CNRS 6302), Univ. Bourgogne Franche-Comté, 21000 Dijon, France; caroline.guigou@chu-dijon.fr (C.G.); sarah.leclerc@u-bourgogne.fr (S.L.); alain.lalande@u-bourgogne.fr (A.L.); alexis.bozorggrayeli@chu-dijon.fr (A.B.G.); 2 Otolaryngology Department, University Hospital of Dijon, 21000 Dijon, France; 3 Medical Imaging Department, University Hospital of Dijon, 21000 Dijon, France

**Keywords:** registration, image-to-patient, head-and-neck surgery, computer-assisted surgery, image-guided, image navigation

## Abstract

Today, image-guided systems play a significant role in improving the outcome of diagnostic and therapeutic interventions. They provide crucial anatomical information during the procedure to decrease the size and the extent of the approach, to reduce intraoperative complications, and to increase accuracy, repeatability, and safety. Image-to-patient registration is the first step in image-guided procedures. It establishes a correspondence between the patient’s preoperative imaging and the intraoperative data. When it comes to the head-and-neck region, the presence of many sensitive structures such as the central nervous system or the neurosensory organs requires a millimetric precision. This review allows evaluating the characteristics and the performances of different registration methods in the head-and-neck region used in the operation room from the perspectives of accuracy, invasiveness, and processing times. Our work led to the conclusion that invasive marker-based methods are still considered as the gold standard of image-to-patient registration. The surface-based methods are recommended for faster procedures and applied on the surface tissues especially around the eyes. In the near future, computer vision technology is expected to enhance these systems by reducing human errors and cognitive load in the operating room.

## 1. Introduction

Surgical navigation systems also known under the general term of computer-assisted surgery (CAS) systems were introduced into routine medical practice more than four decades ago [1]. Today, hybrid operating rooms including computed tomography (CT) scans or magnetic resonance imaging (MRI) are rapidly expanding around the world in all specialties [2]. Based on this technology, minimally invasive surgical procedures and endovascular interventions have been designed [3]. These types of procedures require specific training and collaboration in the fields of imaging and surgery [4,5]. Although these systems represent real progress in many surgical fields, the flow of information provided by them increases the cognitive load of the operators and potentially the risk of human error [6], raising many issues pertaining to safety, reliability, and ergonomics.

Until the end of the 19th Century, the only way to explore the human body’s organs was through invasive procedures [7]. Later, the advent of X-ray imaging by Wilhelm Conrad in 1895 was acknowledged by the medical society and rapidly became the key to the exploration of human anatomy [8]. In the following years, the number of applications for this new technology grew rapidly. However, to establish the correspondence between the image and the patient’s body, physicians had to rely on their anatomical knowledge and mental representation capacities [9]. The first attempts to localize specific anatomical structures based on imaging can be traced back to the late Nineteenth Century [10]. The use of navigation systems has since grown rapidly, especially in the head-and-neck region. At the beginning, these systems used an external frame attached to the target body region. After obtaining an X-ray image including the frame and the concerned body region (generally the skull), the coordinates of the target were calculated, and an instrument was placed on the target using the same frame [10]. In simple scenarios, such as stereotactic brain biopsies, the procedure could be carried out with a quite simple technology. However, in complex surgical procedures requiring the mobility of both the head and the surgical instruments, the introduction of several instruments in the field, and the movement of soft tissues inside and around the target zone, complex and sometimes cumbersome machines (e.g., O-Arm, Medtronics Inc., Minneapolis, MN, USA) became unavoidable. Today, these systems can provide crucial anatomical information during the procedure to decrease the size and the extent of the approach, reduce intraoperative complications, and increase accuracy, repeatability, and safety [11].

**Figure 1:**
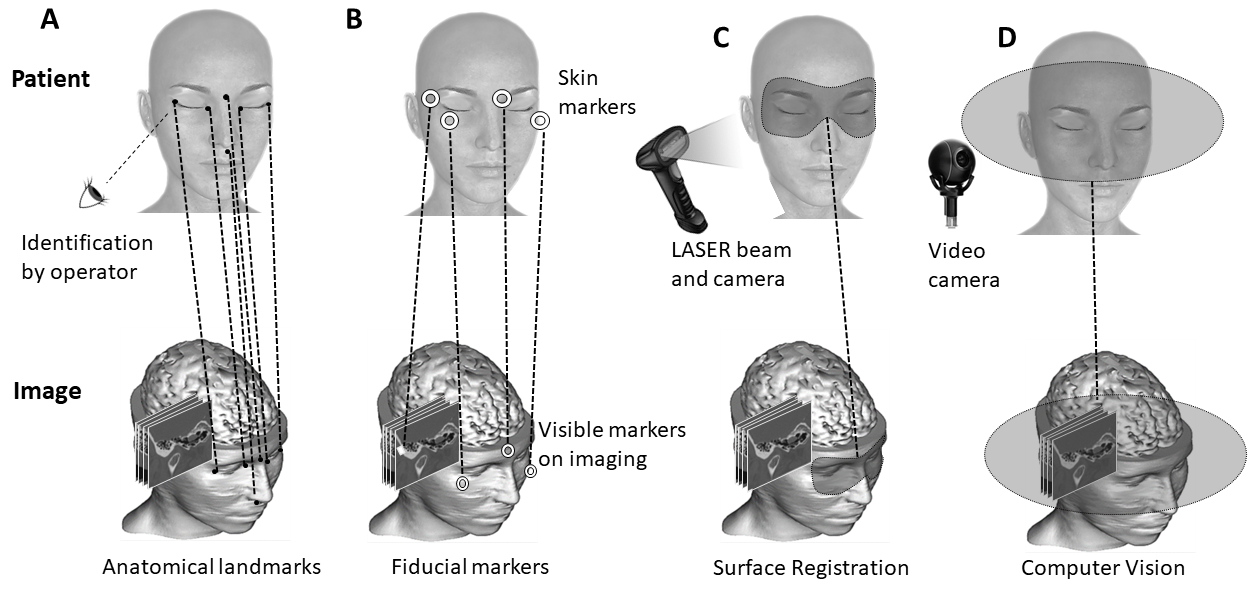
A classification of the registration methods. The upper row represents the intraoperative data (patient’s body) and the lower row the preoperative data (imaging). The dotted lines represent the pairing between the 2 modalities. In the anatomy-based method (**A**), anatomical landmarks are identified and selected by the operator. In the marker-based method (**B**), fiducial markers are fixed to the patient’s head. In the surface-based method (**C**), the red surface on the patient’s face is scanned by a specific instrument. In computer-vision-based method (**D**), the registration zone is captured by a camera.

All CAS systems require an image-to-patient registration as a preliminary step. This step consists of aligning multiple coordinate systems. The two inputs are the target (intraoperative, such as a biopsy needle) and the source (preoperative, such as a brain MRI scan). The process is conducted by transforming the source image to align with the target. Registration methods can be classified based on their characteristics (Figure 1). An overview of these characteristics is provided below:

- Type of input: Preoperative images (e.g., MRI, CT scan, ultrasound (US)) provide information on the deep-seated structures and are generally acquired several hours or days before the procedure. This timing is due to the complexity of image acquisition and processing. Intraoperative imaging equipment (e.g., cone-beam computed tomography (CBCT), intraoperative CT (iCT), fluoroscopy, US, intraoperative MRI (iMRI), endoscopic cameras) or tracking devices with markers connected to the navigation system serve for the initial image-to-patient registration and also to correct navigational errors or the tissue shift during the procedure [12]. Accordingly, data could take the form of either an image or the spatial coordinates of the physical space.
- Transformations: Depending on the types of surrounding tissues, the registration could be rigid or non-rigid (i.e., deformable, local). Soft tissues require a non-rigid registration that considers all local deformations [13], while a rigid registration considers only global transformation and normally requires fewer degrees of freedom and lower computational costs.
- Techniques: In both cases of rigid and non-rigid techniques, the registration can be manual [14], semi-automatic [15], or automatic [16]. Current studies focus on removing the human intervention from the loop progressively and ultimately automating the whole procedure. This enhances the ergonomics while potentially increasing the performance. Today, the most-common scenario consists of a surgeon or a qualified operator conducting a manual registration by selecting a set of corresponding points from the patient’s physical space or the target image based on a qualitative analysis. A semi-automatic procedure employs a program that can assist the registration process to enhance the performance, the accuracy, or the computational cost, but still requires the intervention of an expert. The chain of procedure can include well-known algorithms such as the iterative closest point (ICP), normal iterative closest point (NICP) [17], or even learning-based algorithms [18]. Several other routine CAS systems are based on automatic registration. The process does not require any intervention, but in some cases, invasive external fiducial markers and external tracking devices with bulky sensors attached to the body are required [19].
- Correspondence: Establishing a correspondence between the input data is based on the input modalities and their available features. The three common approaches for determining the correspondence are [20]: segmentation [21], sparse features (i.e., points, edges, objects) [22], or signal intensity (i.e., MRI or US signal, radiological density) [23,24]. Feature-based approaches are known to be less complex in terms of computation, where the transformation matrix could be directly obtained from the correspondence of features between two modalities or by a simple algorithm (e.g., least squares [25]). Features could be physically available (fiducials) or extracted from images with image-processing techniques [26]. However, in cases where the same features are not constantly available in all situations (e.g., block of display, noisy images, anatomical deformations), the reliability becomes an issue. As an alternative, intensity approaches start from the initial default parameters, and through an optimization algorithm, the best model is selected. Here, the key relies on the selected metric, which is at the basis of integrating mutual information from both modalities [27].

Based on this background, image-to-image and image-to-patient are the two types of registration in the medical field. The word “patient” in the latter term refers to either the patient’s physical reference space (real spatial coordinate system) or real-time intraoperative imaging (e.g., endoscopic, CBCT, operative microscope video output). Registration in CAS can, therefore, be referred to as an image-to-patient registration.

Mathematically, the dimension of the source should always be greater than or equal to the target. Since the target (the patient) is always in three dimensions (3D), we expect the source to be also in 3D, thus a 3D-to-3D transformation is expected. As the deformations in the head-and-neck region are considered to be rigid, a minimum of three non-coplanar points or features should be perceived and matched in both inputs. However, in some cases, the target is displayed in two dimensions (2D) by projecting it from a certain viewpoint to a 2D image [28]. Then, an extra point is needed for identifying the projection parameters.

In our work, we will systematically review all procedures of image-to-patient registration in the head-and-neck region and discuss their characteristics, from the perspectives of the method, accuracy, processing time, and invasiveness. To the best of our knowledge, no comprehensive systematic review focusing specifically on the patient-to-image registration has been conducted in the past decade.

## 2. Methods

In this study, we performed a systematic review of the available literature up to April 2022 on the “Pubmed” website (https://pubmed.ncbi.nlm.nih.gov accessed on 12 April 2022) using the following query: “(R & P) OR (R & O & N) OR (R & O & M & N)”, where each term is a list of keywords appearing in the articles’ title. R stands for registration, O for organ, M for modality, N for techniques, and P for procedures as listed below:

- R: register, registration.
- O: auditory, brain, canal, cavity, cephalic vein, cerebellar, cerebral, cerebral, cerebrum, cheek, chorda, ciliary nerves, cochlea, cranial, cricoarytenoid muscle, cricoid, ear, eardrum, eye, facial, fossa, glossopharyngeal, head, hypoglossal, iris, jaw, jugular, laryngeal, larynx, lingual, lip, malleus, mandibular, masticatory, maxillary, meninges, muscles, nasal, nasolacrimal duct, neck, nerve, nose, occipital lobe, ocular, oculomotor, optic chiasm, optic nerve, oral cavity, palate, palpebral, peduncles, pharynx, retina, septum, sinus, sinuses, skull, submandibular gland, teeth, temporomandibular joint, tongue, tooth, trachea, trochlear nerve, tympani nerve.
- M: angiographic, angiography, CT, DPI, fluoroscopic, fluoroscopy, image, imaging, Laser, mesh, modality, MRI, portal image, surface, techniques, TRUS, ultrasound, US. N: 3D to 2D, AR, augmented reality, biopsy, endoscopic, endoscopy, FNA, guided, IGRT, image to patient, image-to-patient, implant, implantation, interventional, intra, intraoperative, intra-operative, invasive, macroscopic, macroscopy, navigation, non-invasive, radiosurgery, radiotherapy, real time, shift, surface, surgery, surgical, video.
- P: angle tumor, apex, cataract, cavernous, cerebellopontine, cervical spinal, clivus, cochlear, craniotomy, gamma-knife, glaucoma, jugular, keratectomy, labyrinthectomy, labyrinthectomy, lasik, macula, myringotomy, neuroendoscopy, olfactory, ophthalmology, otologic, petrous, photorefractive, PRK, retinopathy, septoplasty, sinusitis, stereotactic, tracheostomy, turbinoplasty.

The initial search yielded 483 studies. After removing duplicates, reading the abstracts for appropriateness in terms of scope, and including 20 additional articles through cross-referencing, a total of 113 studies were included in this review.

## 3. Registration Methods

The registration methods (Figure 1) were classified based on the type of input data and the acquisition technique: anatomy-based, surface-based, marker-based, and computer-vision-based.

### 3.1. Anatomy-Based Methods

**Figure 2:**
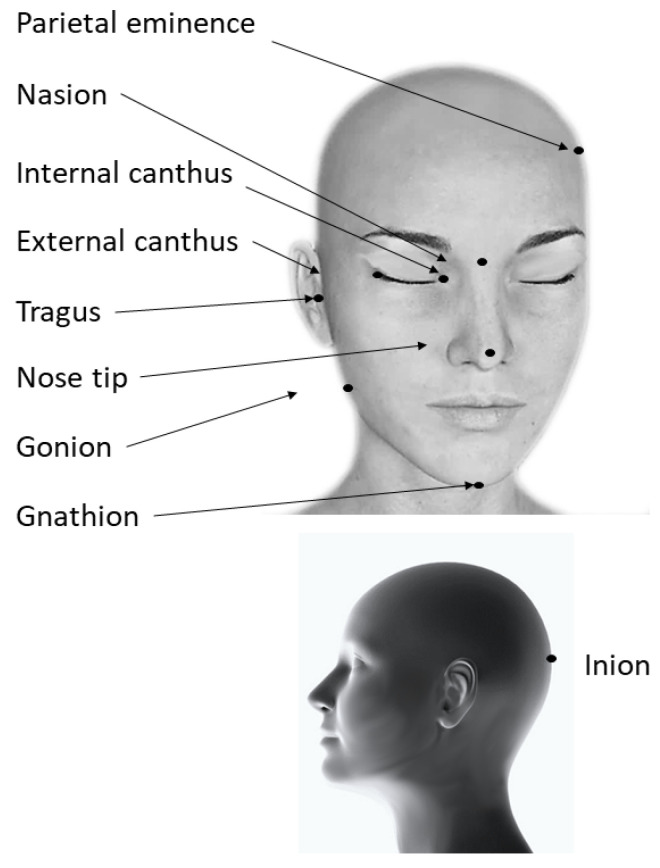
Landmarks used in anatomy-based methods for registration.

They are basically a pair-point matching. Characteristic points are specific anatomical landmarks that could easily be identified in both the image space and the patient space with minimal skin shift. Common landmarks are the tip of the nose, nasion, both canthi, tragi, parietal eminence, and inion [9,29] (Figure 2). Teeth can also be used as landmarks: the mesiobuccal cusps of the first molars on both sides, the mesial point of the incisal edge of the upper incisor, and the canine cusp were used in a study utilizing digitally reconstructed models [30]. Depending on the procedure, some landmarks may be hard to reach due to the head position. While most of them are accessible in a supine position, only a few are reachable in a lateral position or even fewer in a prone position.

Marking the landmarks on an image requires the placement of a marker on the adequate 2D slice when dealing with 2D images or crosshairs on each plane for more precision in the case of 3D images [31]. Marking in a patient physical reference frame is achieved via a tracked pointer device. By pairing the two sets of landmarks, the coordinate systems can be unambiguously registered. There are several algorithms to calculate the transformation between two spaces from corresponding point pairs [32,33,34]. The anatomy-based method has been reported to show lower accuracy in comparison to other methods. However, it is known for its simplicity, minimal invasiveness, and low cost.

### 3.2. Surface-Based Methods

These methods work by scanning the surface of the face to collect a large set of points forming the geometry of the area. The points are matched to a corresponding set of points extracted from the preoperative imaging. Unlike other methods, there is no direct correspondence between the points. Instead, algorithms aim for the best transformation that aligns both sets. ICP is one of the most-popular algorithms for such a purpose. The algorithm works iteratively, each time estimating the transformation that minimizes a cost function determined by a defined distance between points [35,36].

Optimal regions for registration are the ones with a minimal skin shift (along the nasion, around the orbits, the forehead, and the nose). Evidently, this method is not feasible in a prone position since remarkable landmarks are not accessible to the external cameras [37].

Initially, stationary intraoperative systems were developed to acquire skin surface points. Marmulla et al. proposed multiple systems of this kind based on projecting and capturing an optical signal [38,39]. Beams from a projector (such as a video beamer) fixed above the patient’s head, or laser beams, were projected on the skin and captured by cameras within the specified frequency range of the light.

Later, mobile systems were introduced. The majority of these systems operated contactless by projecting the laser lines (Z-touch, BrainLAB, Heimstetten, Germany). Each line ends with a dot on the patient’s skin. The laser reflections are detected by the infrared cameras of the navigation system placed around the patient’s head, and their positions are exploited for the registration. Alternatively, the distance to each laser dot is measured by the handheld tool itself, therefore eliminating the need for an external camera to capture its position [40]. However, an active optical tracking system, using light-emitting diodes attached to the tool and external infrared cameras, are used to track the position of the registration tool. The tracking of the registration tool establishes the geometrical relation between the system and the patient. In a more-complex surface-based method, several camera systems were designed to capture both image and depth information along the z-axis. Lee et al. [41] reported four commercially available systems using such a method: The GALAXY (LAP Co., Lüneburg, Germany), IDENTIFY (Varian Co., Palo Alto, CA, USA), C-RAD products (C-RAD Co., Uppsala, Sweden), and The Align RT (vision RT Co., London, UK). These systems rely on either a laser camera, time-of-flight camera, structured light, or stereo cameras to acquire the depth information. Simpler surface contact tools were also used to scan skin surface points (e.g., Soft-touch, BrainLAB, Heimstetten, Germany, or Digipointeur, Collin SA, Bagneux, France).

### 3.3. Marker-Based Methods

These methods rely on markers attached to the patient’s tissues and can be either invasive or non-invasive. In both cases, the markers or fiducials are clearly identifiable on both modalities (preoperative and intraoperative images). They are localized easily and, in some systems, automatically. The detection of markers in preoperative images can be simply achieved by thresholding [42]. In the patient’s physical space, points are localized by touching the fiducial marker with a tracked probe [43] or an optical tracker [44] (Hx40; Claron Technology Inc., Toronto, ON, Canada).

Examples of non-invasive markers are stickers, headbands, dental splints, and detachable markers. Headbands (e.g., BrainLAB headband, BrainLAB, Heimstetten, Germany) and stickers (e.g., Digipointeur, Collin SA, Bagneux, France; BrainLAB 960-991 Medtronic Disposable Fiducial Markers, Heimstetten, Germany) on the patient’s forehead are easy to install and inexpensive [45]. They are attached to tissues with minimal shift such as bone by screws, biocompatible glue, or any other adhesive material. The markers should remain in place from the time of the preoperative imaging until the registration in the operating room. Yet, the skin movements during the imaging and registration may still induce millimetric errors. Detachable markers can be customized to the patient’s anatomy preoperatively. Their shape does not change until the day of the operation, thus there is no requirement for the markers to be placed between the time of preoperative imaging and the day of the procedure. A common marker based on this concept is the Thermoset facial mask used for immobilizing the patient’s head-and-neck for radiotherapy [46]. More on this subject will be discussed in the invasiveness section.

Invasive markers placed under local anesthesia in the skull bone have the advantage of no tissue shift, but their invasiveness represents a limitation [47]. Titanium screws are preferred because they present a low risk of allergy, are biocompatible, and cause no inflammation [48].

### 3.4. Computer-Vision-Based Methods

These methods do not require any marker, and unlike surface-based methods, the patient’s space is captured by a real-time video. Basic acquisition tools are a video feed from an endoscope [49], a microscope [50], or a stereo camera [51]. For instance, in a procedure of deep brain stimulation, Zagorchev et al. [52] used a geodesic photogrammetry system (Electrical Geodesics Inc., Eugene, OR, USA) for the identification of the electrode positions on the scalp surface. The system relied on 11 cameras mounted on a polyhedron mount. It could compute the location of the electrodes by stereovision principles combining the position of each camera and the principles of perspective [53]. In a similar manner, Chang et al. [51] used two stereo cameras for 3D modeling. The patient’s physical space was derived from a pair of images. For smaller surgical fields, more-complex tools were utilized. Gurbani et al. [54] implemented a system for cochlear implantation with a fiber optic rotary probe. The method was based on scanning the inner surface of the cochlear canal using the probe attached to a microrobot (EyeRobot2, CISST ERC Johns Hopkins University, Baltimore, MD, USA) [55]. The probe then acted as a highly accurate distance sensor, allowing for surface detection. To deal with brain shift in neurosurgical procedures, Jiang et al. [56] were able to measure the texture of the brain surface with a phase-shift 3D surface measurement system [57,58]. The brain surface was scanned by projecting phase-shifted patterns in both the horizontal and vertical directions, while the phase-shift pattern was recorded by a camera. Subsequently, the camera captured the 2D texture image.

The inputs (intraoperative and preoperative data) of computer-vision-based methods are correlated either through a similarity metric or a feature extraction followed by feature mapping. However, it is common to preprocess the input data as a preliminary step. Preprocessing involves image reconstruction or rendering. In a study that evaluated a navigation system for sinus surgery, Burschka et al. [59] used a monocular camera to obtain a 3D reconstruction of the target region and used it as an input to a principal-component-analysis (PCA)-based algorithm, which registered the reconstructed video data to the CT data. The method was inherited from a previously implemented vision-based inertial system for mobile robots. Another approach to preprocessing data is to use image rendering while attempting to apply the appropriate metric [60]. By combining the information on camera pose to the video feed via an optimization algorithm, a rendered image (virtual endoscopic vision) with maximum similarity to the real 3D endoscopic image can be obtained as reported by Otake et al. [61]. This rendering algorithm, called iso-surface volume rendering, was implemented on a graphics processing unit (GPU) for faster calculation and more fluid images. The similarity metric was based on the normalized cross-correlation (NCC) between the gradients of the two images. The concept of the GPU-based rendering method was also applied to CT scan data by using an algorithm based on a structural similarity metric [62]. The system searched in the CT scan data for the virtual camera pose that produced a simulated view that best matched the real video image. These projects, which take advantage of the considerable image-processing capacity of the GPUs in real-time, will be expanding in the near future.

## 4. Accuracy

Accuracy enhancement in the surgical procedure stands out as one of the main objectives of the CAS. In the head-and-neck region, the accuracy depends on the region. In stereotactic procedures that involve a variable amount of brain shift, an accuracy above 2 mm is considered as unacceptable [63,64]. An even lower threshold is set for microsurgical interventions. One prominent example for this high-accuracy requirement is cochlear implant surgery, where the accuracy shall not exceed 0.5 mm [65]. Indeed, the average width of the posterior tympanotomy separating the facial nerve from the rim of the external auditory canal and through which the approach is drilled is 4.7 mm [66]. In cataract surgery, the threshold of clinical acceptance drops to the range of 20 to 100 µm [67,68].

**Figure 3:**
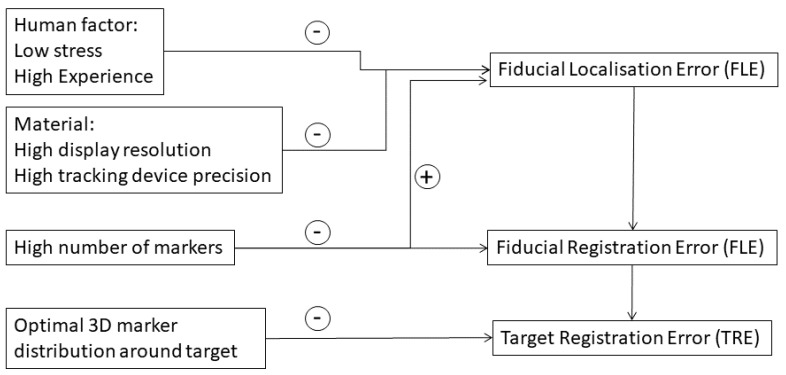
Metrics and factors influencing the target registration error (TRE) in anatomy- and marker-based methods. the sign − indicates that the corresponding factor decreases the error. The sign + indicates that the corresponding factor increases the error.

Whatever the adopted method, the target registration error (TRE) is the common measure of accuracy and the only metric to estimate the reliability of the intraoperative information [69]. However, in particular methods, other notations of error metrics emerge before estimating the final registration output TRE. Fiducial localization error (FLE) and fiducial registration error (FRE) could assist in the evaluation of the registration method (Figure 3).

### 4.1. Target Registration Error

The TRE is defined by the distance between the actual and the estimated target position after the registration. A principle applies for all methods: TRE increases with the distance between the registration (fiducials or features) and the target areas [70,71,72,73,74]. Eggers et al. [75] conducted a study to test whether a maxillary template is sufficient for image-guided cranial surgery. The TRE increased from 1.5 mm in the anterior to 3.26 mm in the lateral skull regions since the maxillary template used in the registration was fixed in the frontal region. In the same context, Marmulla et al. [76] were able to improve their surface-based method and approach the results of conventional marker-based methods in the lateral skull base surgery by scanning the auricle. Reducing the distance between the registration area and the target reduced the chances of obtaining an inaccurate registration matrix. The main disadvantage of using the pinna was that the cartilage is easily deformable, and care should be taken during CT scan acquisition to avoid contact between the headrest and the pinna, which will distort the landmarks. In another study, Bozorg Grayeli et al. [77] came to the same conclusion when placing a titanium screw behind the auricle in the temporal bone. Using a screw in this location as a fiducial marker in addition to the midface skin registration significantly increased the precision of the navigation in the lateral skull base. Opposite this, only one study [78] claimed that the TRE was the same in the anterior and lateral skull regions. The method relied on a dynamic reference frame attached to a skull phantom, and markers were detected automatically through infrared technology.

Another factor that directly affects the TRE is the spatial distribution of the markers. It is recommended to avoid collinear placement of the markers and keep them as far apart as possible [79]. In other terms, the aim is a wide distribution of the markers with minimal redundancy in the x, y, and z axes [80,81].

In pair-point matching methods (anatomy- and marker-based), apart from the previously mentioned parameters, the TRE is influenced by several other factors related to the marker localization and number and the alignment method and human factors such as expertise, stress level, and fatigue (Figure 3). For these factors, the FLE and FRE are interesting metrics to analyze the system in addition to the TRE.

### 4.2. FLE

The FLE is the distance between the true position of a fiducial marker and its measured position. Factors that influence the FLE in the image coordinate space include the shape and the size of the marker, the voxel dimensions of the image (error decreases as the marker size/voxel dimension ratio increases), the digital properties of the image (spatial and intensity quantization), the signal-to-noise ratio of the image, the marker’s contrast relative to its background in the image, the geometrical distortion of the image, and the localization method [43,82].

In the anatomy-based methods, the localization of the markers in the patient’s physical space depends on the operator’s ability to identify them and match them with the corresponding point on the preoperative image [83]. Kral et al. [84] conducted a study on four residents in the same year of postgraduate training with no experience in CAS. They performed pair-point registrations on five anatomic specimens. The accuracy increased with the repetition of the procedure, suggesting the effect of the surgeons’ experience.

In the marker-based methods, locating the markers relies on the detection tool (e.g., probe). The tool should bear attached markers to be tracked by the CAS, and this can introduce potential errors into the chain of events. Knott et al. [43] showed that attaching a rigid tool to the probe with a number of well-spatially distributed markers for tracking resulted in a 0.1 mm mean transformation error (error in estimating the position of the registration tool by the navigation system) in comparison to the standard probe. However, an increase of 0.37 mm in the tool tip localization error (tracing accuracy of the tool tip) appeared to be due to the poor ergonomics caused by the bulky rigid tool attached. Furthermore, Gerber et al. [50] were able to achieve an excellent accuracy of 0.1 mm by eliminating the human intervention and the use of robotic fiducial localization (Table 1). Fiducial markers were located on the patient by an automatic robot-based tactile search within the head of a standard surgical screw.

### 4.3. FRE

The FRE is the distance, after registration, between the fiducial marker positions in the preoperative image used in the registration process and their corresponding point sets on the patient coordinate. The localization and number of fiducials have the greatest effect on the FRE (Figure 3). An increased number of fiducials (N) will lead to a higher value of the FRE as it is more difficult to align multiple markers [79]. However, this same increase in N might drop the value of the FLE since more markers used in the registration process will reduce the effect of localization error [80] (Figure 3). Nevertheless, extending the time for acquisition during the surgery might raise the surgeon’s workload and lead to an increase in the FLE. In a study attempting to quantify the registration parameters that influence accuracy, Chu et al. [85] suggested that using five fiducial markers is the optimal trade-off between the registration time and the accuracy.

The FRE is the only accuracy indicator that can be measured during the registration process, but it should be pointed out that the level of the FRE might be misleading for the surgeon since it does not entirely reflect the accuracy of the CAS around the target [70,79]. In fact, the FRE is independent of the spatial distribution of the markers and clearly does not consider the distance between the target and the markers serving for the registration (Figure 3).

**Figure 4:**
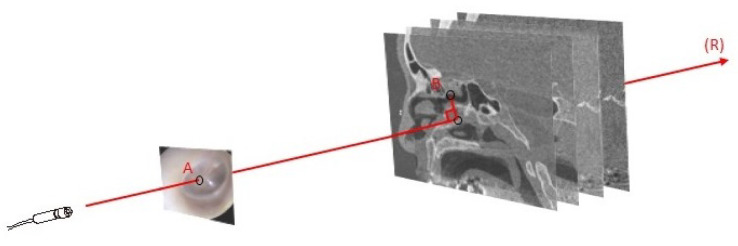
Target registration error between 2D and 3D modalities. B is the target in the 3D modality. A is the registered target in the 2D modality. A ray (R) is issued from the center of the camera and passing through A. Error is measured by the perpendicular distance from B to R.

In systems where one of the inputs has two dimensions, which is common in computer-vision-based methods, the conventional TRE is replaced by a projective TRE (Figure 4). To measure the distance between Point A in the 2D registered image and Point B in the 3D image, a ray R is issued from the center of the camera and passes through A. The error is then defined by the perpendicular distance between B and the ray R. In the case of a perfect registration, the line would pass exactly through B, resulting in an error equal to zero [26,61,86,87].

Table 1 and Table 2 summarize publications on image-to-patient registration with the highest accuracies (lowest TRE) in different procedures. Publications on otological procedures report TREs below 0.5 mm, while CASs used for rhinology and neurosurgery show TREs less than 1 mm. However, it is important to note that this accuracy depends on several factors. As mentioned before, the distance between the target and the markers is a crucial factor in the TRE. In the tables, this factor can be estimated by the distance between the two columns “registration landmark” and “target landmark”. Another factor is the operating environment, where working in a realistic scenario is related to a higher TRE probably due to stress and time constraints. For instance, the TRE of the same CAS increased from 0.69 [88] and 0.8 [61] to 1.19 and 1.97, respectively, when applying the same method on a plastic head or a cadaver versus a patient.

**Table 1:** Top computer-assisted surgery settings in terms of accuracy in head-and-neck, excluding neurosurgical procedures.

Study	Procedure	Registration Landmarks	Test Subjects	Method	Description or Commercial Name	Target Landmarks	TRE (STD) [Min–Max]	Time
Gerber et al. [50]	Otology	Outer surface of the mastoid	1 plastic temporal bone *n* = 32	MB	Image-guided robotic microsurgery on the head	Cochlea and round window	0.07 mm (0.019)	<4 min
Zhou et al. [89]	Otology	Mastoid bone	13 patients	SB	Contact surface matching	Mastoid surface	0.16 mm (0.09)	3 min
Zhou et al. [89]	Otology	Mastoid bone	13 patients	SB	Contact surface matching	Round window	0.23 mm (0.1)	3 min
Lavenir et al. [83]	Otology	Cochlea in HFUS images	6 cadaveric guinea pig cochleae	AB + CVB	Registering micro CT scan to HFUS	Cochlear structures determined on 3D images	0.32 mm (0.05)	NS
Schneider et al. [90]	Otology	Middle ear, auditory canal, mastoid cortex	2 specimens	AB	Pair-point matching	Middle ear, auditory canal	0.51 mm (0.28)	3.8 min
Hauser et al. [88]	Rhinology	Nasion, outer ear, upper teeth	1 plastic head *n* = 160	MB	1 dental face bow	3 markers intranasal + 1 marker extranasal near the nose	0.69 mm (0.2)	NS
Otake et al. [61]	Rhinology	Sinus endoscopic rendered image	1 cadaver *n* = 7	CVB	Rendering-based video CT	Sinus (2D to 3D TRE)	0.83 mm	4.4 s
Brouwer de Koning et al. [91]	Maxillofacial	Teeth	1 phantom *n* = 45	MB	3D-printed dental splint	Mandibular	0.83 mm [0.70–1.39]	>90 min
Broehan et al. [67]	Ophthalmology	Retinal vessels from ophthalmic microscopeframes	10 patient video sequences	CVB	CAS for laser photocoagulation system	Retinal vessels on ophthalmic microscope	23.2 μm (18.8)	Real time
Reaungamornrat et al. [92]	Laryngology	Region of tongue extending to hyoid bone on CBCT	1 cadaver (25 images)	CVB	Deformable image registration for base-of-tongue surgery	Tongue surface	1.7 mm (0.9)	5 min

Methods are grouped according to their procedures. AB = anatomy-based registration method, MB = marker-based registration method, SB = surface-based registration method, CVB = computer vision-based registration method, HFUS = high-frequency ultrasound, CBCT = cone-beam computed tomography, NS = not specified.

**Table 2:** Top computer-assisted surgery settings in terms of accuracy in neurosurgical procedures.

Study	Procedure	Registration Landmarks	Test Subjects	Method	Description or Commercial Name	Target Landmarks	TRE (STD) [Min–Max]	Time
Fu et al. [74]	Skull-base surgery	Intraoperative X-ray images	1 head-and-neck phantom *n* = 49	CVB	Intensity-based intraoperative X-ray to CT registration in radio surgery	Skull surface	0.34 mm (0.16)	<3 s
Ledderose et al. [93]	Skull-base surgery	Teeth	1 cadaver *n* = 20	MB	Dental splint for lateral skull surgery	Left lateral skull base	0.55 mm (0.28)	NS
O’Reilly et al. [94]	Skull-base surgery	Skull surface by HFUS	5 cadaveric human heads	CVB	Registration of human skull CT data to an ultrasound treatment space	Skull surface	0.9 mm (0.2)	NS
Marmulla et al. [76]	Skull-base surgery	Auricle	10 patients	SB	Laser Surface registration for lateral skull-base surgery	Periauricular bow	0.9 mm (0.3)	2 s
Gooroochurn et al. [95]	Skull-base surgery	Ear tragus, canthi	1 artificial skull	CVB	Facial recognition applied to registration of patients in the emergency room	4 landmarks near the canthi and the ear tragi	0.98 mm [0.52–1.31]	NS
Saß et al. [96]	Skull-base surgery	Skull surface	30 patients	MB	Frameless stereotactic brain biopsy	Skull surface	0.7 mm (0.32)	36 min
Xu et al. [97]	Deep brain stimulation	Skull surface	38 patients	MB	Registration in deep brain stimulation using ROSA robotic device	Implanted rod	0.27 mm (0.07)	NS
Hunsche et al. [98]	Deep brain stimulation	2D X-ray images	15 patients	CVB	Intensity-based 2D to 3D registration for lead localization	Implanted rod	0.7 mm (0.2)	<20 min
Jiang et al. [56]	Brain surgery	Brain texture surface + camera images	5 porcine brains	CVB	Non-rigid registration integrating surface and vessel/sulci features	Brain surface	0.9 mm (0.24)	340 s

Methods are grouped according to their procedures. MB = marker-based registration method, SB = surface-based registration method, CVB = computer vision-based registration method, HFUS = high-frequency ultrasound, NS = not specified.

## 5. Processing Time

Time is essential in the process of image-to-patient registration. Long CAS setups expose clinicians to stress, increase their cognitive load, and divert them from their clinical tasks. Visuo-tactile perceptions tend to be vague especially if the surgery lasts longer than expected [85]. In contrast to offline registration tasks, which are conducted outside the operating room, the image-to-patient initial registration in the operating room should not exceed 5–10 min [14]. Longer durations will result in a significant increase in the workload of preparation and the checklist before surgery.

For re-initiating the registration during the operation, the acceptable limit is even lower since surgical constraints (e.g., bleeding, vascular clamping delay) or stress do not allow a five-minute break. One or two minutes may be considered acceptable during a procedure if it is not repetitive. However, in some procedures, iterative registrations may be required during the procedure, and this is considered as a challenge, especially with the tissue shift and the patient’s movements during an operation. Data from the initial registration may not be valid and exploitable for recalibration [99]. In this type of scenario, interruptions of 1 or 2 min in the procedure may dramatically affect the ergonomics if they are frequent.

Comparisons between the anatomy-based, the surface-based, and the marker-based methods [31,85,100] suggest that the surface-based methods are generally the fastest and that more human intervention is related to slower registration [101,102]:

- *Surface-based* methods require shorter registration procedures due to their simplicity in both the setup and process of acquisition. In two studies on patients undergoing endoscopic sinus surgery, scanning with optical and electromagnetic devices required an average of 3 min for the equipment setup and less than 50 s to perform the registration [101,102].
- *Anatomy-based* methods are highly dependent on the perception of the operator. The process might be repeated several times to achieve the optimal accuracy and is, consequently, longer [100].
- *Marker-based* methods require more-complex and longer setups and can be further prolonged by sophisticated labeling and marker fixation into the bone [103]. In case of an inexperienced surgeon, an additional 15–30 min may be necessary for the overall process [104]. Even for non-invasive fiducial labels, the environment setup for fixing the markers might take several minutes or hours [91]. In a method described by Matsumoto et al. [105], building a customized template of the patient required a patient clinical visit 2 weeks before the surgery and, hence, a more-complex logistical organization than other registration routines. This method will be described with more details in the invasiveness section.
- *Computer-vision-based* methods require the least amount of human intervention or reliance. The overall process might take several seconds or several minutes when running on commercial central processing units (CPU) [106]. With the expansion of GPUs for these applications, heavy calculations could be performed instantly [61].

## 6. Invasiveness

In almost all medical specialties, there is a growing trend towards the development of less-invasive and safer surgical procedures [88]. Of all the available methods, the marker-based ones raise the most-significant issues regarding invasiveness. At best, skin markers can cause slight discomfort (headbands) or allergic reaction (adhesive skin markers).

In the conventional marker-based methods, one or several invasive external markers (e.g., bone screw markers, stereotactic frame, Mayfield clamp) are fixed to the bone through soft tissues. Even though invasive markers in bone are more reliable [107], they potentially induce complications and pain.

**Figure 5:**
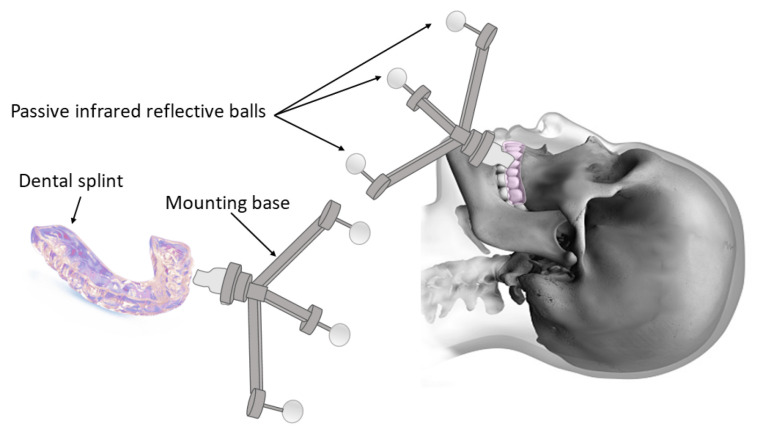
Frame used in marker-based methods fixed on a dental splint.

The methods listed below have been proposed to reduce the invasiveness of the registration systems:

- One common approach is attaching the markers to the upper teeth or the maxillary bone as reference points [30,88,108]. The most-widely implemented systems were designed with a reference frame to be mounted on a dental splint or a mouthpiece (Figure 5) [75,91,109,110,111,112]. For instance, a registration tool tightly attached to the upper teeth by means of a silicon rubber splint bearing automatically recognized markers was developed and validated for cochlear implantation surgery [65]. Although such methods eliminated the complications of penetrating the skull, their accuracy was slightly below invasive approaches [113,114].
- Alternatively, customized facial masks were conceived of, allowing a relatively precise registration without using an invasive marker. Ford et al. [115] proposed a facial mask (manufactured and provided by Xomed Corporation, Jacksonville, FL, USA) made of a radiolucent, low-melting-temperature polyester with ten radiopaque fiducial markers embedded permanently into it. This mask was used for a CAS dedicated to sinus surgery. The mask is placed in warm water until it is deformable enough then held on the patient’s face until it solidifies into shape. It fits on rigid structures with multiple contact points such as the frontal, nasal, maxillary, and parietal bones, and it can be securely strapped at the back of the head. However, the presence of a facial mask reduces the access to many facial and nasal regions and cannot be applied to many procedures. In a similar manner, Hubley et al. [116] used a facial thermoplastic mask for Gamma knife radiosurgery. It was applied for immobilization during an onboard CBCT imaging system to define the stereotactic space. Similarly, Chen et al. [117] used a six-marker thermoplastic facial mask for an image-to-patient registration in stereotactic radiofrequency thermocoagulation.
- In contrast, using a headband in brain surgery as an alternative for invasive pins and skull posts does not appear to be a practical solution since they are easily displaced intraoperatively and these unwanted movements reduce the accuracy of the CAS [108,118].
- Surface template-assisted marker positioning (STAMP) is another marker-based method that works by building a template of the bony surface of the patient’s head from a preoperative CT scan [105]. Virtual markers are created on the CT scan and then transferred to the patient’s head template, which is manufactured by 3D printing. Virtual markers are represented by holes in the template. Intraoperatively, a sterile template is placed on the patient’s head, and the positions of the virtual markers are marked by a pen through the holes, establishing the correspondence between the CT scan and the patient’s head.
- Another way of securing the markers without tissue penetration is to place them in the nasal cavities. For an auditory brainstem implantation, the fiducial markers were mounted on a titanium mesh connected to a stent, which was placed through the nostrils into the rhinopharynx. This device was initially commercialized for the treatment of sleep apnea (AlaxoStent, Alaxo GmbH, Frechen, Germany) [119,120].

## 7. Discussion

Image-to-patient registration is the initial and a crucial step of every CAS. Initially, marker-based methods were generalized as the most-robust strategy. In the 1990s-to-early-2000 period, this method was enhanced by incremental improvements. The effects of fiducial marker configuration, localization, and registration sequence on the accuracy registration were refined [80,121]. Less-invasive marker-based methods utilizing detachable masks or frames based on dental splints were developed (Figure 5) [93,122]. However, the marker-based methods still require imaging after the fiducial marker placement in addition to the imaging performed for diagnostic purposes, and this signifies potential additional irradiation.

Later, surface-based methods were introduced. They were able to circumvent the additional imaging, to reduce both invasiveness and procedure time. However, these advantages came at the cost of a lower accuracy [56,76,123,124]. The surface-based methods rely only on the external shape of the body surface, which raises the issue of low reliability during the procedure [125]. More recently, the computer-vision-based methods were conceived of, benefiting from the development of powerful GPUs. One of their greatest advantages is their real-time processing with no need for a complex setup or operator expertise. These characteristics are particularly suitable in the emergency surgical room and offer the possibility to easily reinitiate the process during the intervention if needed [95]. However, the reliability of these methods is still questionable. The computer-vision-based methods need to be challenged with a “noisy” environment (e.g., rapid movements, bleeding, anatomical distortions, partial obliteration of the view) in every possible situation.

In the last decade, many teams have investigated the idea of using the preoperative imaging techniques intraoperatively. MRI and CT scan were brought to the operating rooms and were integrated with the CAS. Studies on iMRI are limited [73,126,127]. This is principally due to not only the cost of the system, but also to the significant technical constraints related to the powerful magnetic field generated by the MRI inside an operating room full of metallic instruments. Other factors are the lower spatial resolution of the MRI, difficulty observing bony structures, and MRI contraindications related to already implanted metallic or magnetic devices in the patients (e.g., pacemakers). iCT, which does not have these limitations, progressed faster and better toward the commercial industry. Several reports showed that iCT provided an accuracy for CASs similar to the conventional methods [128,129], while reducing radiation and saving time and hospital resources [130,131]. However, iCT faces other challenges before it can be widely used for head-and-neck CAS. The risk of exposure to frequent radiation for the surgeons, the loss of time caused by multiple CT acquisitions [132], the cumbersome material, which imposes ergonomic adaptation, and the risk of shifting from the routine of reliable conventional methods into other systems have to be studied for each surgical scenario separately with its pros and cons.

Irradiation is a general concern when dealing with CAS and minimally invasive surgery [133]. An average of two to three CT scans per procedure increases the risk of radiation-induced carcinogenesis [134] and cataracts [135]. Many teams have published optimized protocols to reduce irradiation for both registration and navigation [98,136]. In deep brain stimulation surgery, replacing the conventional CT scan by a 3D fluoroscopy leads to a five-fold decrease of irradiation while maintaining a similar accuracy and process duration [128]. The use of iCT in stereotactic brain biopsy instead of a conventional preoperative CT scan to locate the fiducial markers reduced the irradiation up to eight times [96,137,138].

In our review, methods relying on image processing were classified as computer-vision-based techniques. The fact that some of them do not involve computer vision is debatable. However, it is certain that they share the same characteristics in that they rely solely on markers or surface scanning and that the input data have to be processed or interpreted by a computer program before the registration. In the field of image-to-patient registration and in contrast with other medical imaging domains, deep learning is not common [139]. So far, it cannot compete with the state-of-the-art methods [28,140], but will certainly improve in the coming years. Obstacles in this domain are numerous, and among these, the most-prominent are the lack of large databanks, anatomical variations, scenario variations, and noisy images due to the disease or bleeding.

Almost all registration methods in this review were based on a rigid transformation exploiting the rigid anatomical structures (i.e., bones) in the head region. Furthermore, 51 studies out of the 113 selected described their target region as the head without specifying the exact area. Consequently, the majority of the proposed methods offered common characteristics such as the fiducial markers in bones, the anatomical landmarks in the facial area around the eyes, the intraoperative modalities, and the rigid transformation. In fact, several systems designed for a specific procedure hold significant potential to be used in other procedures in the head-and-neck region, given that the proximity between the registration area and the target area remains minimal. One major exception is the brain tissue [141], where non-rigid deformations may exist especially in the case of intracranial tumors and during their surgical removal [142]. In this case, intraoperative ultrasound (iUS) was commonly used in combination with preoperative MRI to account for the tissue shift. This combination required image-processing techniques to determine the brain deformations and update the preoperative MRI. This strategy has its limits. The non-rigid image-based transformations would not consider the mechanical properties of the anatomical structures depicted in the MRI image and may yield non-physically coherent deformation fields. To ensure the plausibility of the predicted deformation field, biomechanical models have complemented the image-based methods to ensure a realistic computer simulation of the brain deformation. Some authors proposed a segmentation of the brain vessels followed by a linear registration and a refinement by the thin plate spline (TPS) transform [143]. Here, the TPS technique uses several control points (segmented vessels in the iUS) to transform a space (brain on MRI) by mathematical smoothing and interpolation. It has the advantage of a relatively low computational cost, but it lacks precision in a region where tissue properties are not homogeneous (cortex, small and large vessels, ventricles, etc.). In most practical cases, brain deformation models employ the finite element method, which generally includes more-detailed information of the brain tissue including the physical properties and the adjacent structures [73,144,145,146,147,148].

All proposed registration methods have their limitations in the emergency room, where time constraints and stress levels are high. In addition, factors such as patient condition and the availability of surgical skills and technologies should also be taken into consideration for CAS indications [149].

## 8. Conclusions

In conclusion, our work established that all systems are built on trade-offs between the performance, on the one hand, and the setup complexity and invasiveness, on the other, and that different parameters should be privileged depending on the scenario. This systematic review showed that the invasive marker-based method is still considered as the gold standard for image-to-patient registration. The surface-based methods are recommended for faster procedures and applied on the surface tissues especially around the eyes. Computer-vision-based methods combined with artificial intelligence emerge as the future of image-guided procedures leading to lighter, faster, more-precise, and user-friendly systems. They will potentially allow less-experienced physicians to perform interventions in a more-reliable and safer environment. Additionally, certain systems designed for a specific procedure hold significant potential to be used in other procedures in the head-and-neck region, given that the proximity between the registration area and the target area remains minimal.

## Data Availability

All information presented in this review is documented by the relevant references.
